# Stannous Fluoride in Toothpastes: A Review of Its Clinical Effects and Likely Mechanisms of Action

**DOI:** 10.3390/jfb16030073

**Published:** 2025-02-20

**Authors:** John W. Nicholson

**Affiliations:** 1 Bluefield Centre for Biomaterials Ltd., Kemp House, 152-160 City Road, London EC1V 2NX, UK; john.nicholson@bluefieldcentre.co.uk; Tel.: +44-(0)979-8379; 2 Dental Physical Sciences Unit, Institute of Dentistry, Barts & The London School of Medicine and Dentistry, Queen Mary University of London, Mile End Road, London E1 4NS, UK

**Keywords:** stannous fluoride, toothpaste, clinical effects, caries, hypersensitivity, gingival health, structure, aqueous solutions

## Abstract

This article reviews the topic of stannous fluoride as an anti-caries additive in toothpastes. It is based on a literature survey carried out using Science Direct, supplemented by information from PubMed. The keywords used were stannous fluoride, toothpaste, clinical effects, caries, hypersensitivity, gingival health, structure and aqueous solutions. The initial searches covered the period 2015–2024 and identified 57 references. Older references cited in these papers, and also papers already known to the author, were also included. The information thus obtained shows that stannous fluoride has three main effects, namely, reduction in the viability of the oral biofilm, increase in remineralisation of the hydroxyapatite tooth mineral and occlusion of dentinal tubules leading to reduced hypersensitivity. Stannous fluoride was shown to be the most effective of all the fluoride additives used in toothpastes. In much of the dental literature, this is attributed to the effects of Sn^2+^ ions. However, as has been shown extensively in the wider scientific literature, free Sn^2+^ ions do not occur in aqueous systems. Rather, the initial products of the dissolution of SnF_2_ is undissociated, hydrated SnF_2_ and SnF^+^ ions. These gradually exchange fluoride to form Sn(OH)_2_ and Sn(OH)^+^. Their likely mechanism of action based on their toxicity towards oral micro-organisms and their interaction with hydroxyapatite is discussed.

## 1. Introduction

This review article is based on a literature survey carried out using Science Direct and supplemented with additional information identified from PubMed. The keywords used were stannous fluoride, toothpaste, clinical effects, caries, hypersensitivity, gingival health, structure and aqueous solutions. The searches covered the ten-year period 2015–2024 and identified some 57 references published in this time interval. This review was augmented using older references cited in these papers, as well as papers already known to the author. In this way, relevant literature from the last 65 years was identified and included in this article. The clinical effects of stannous fluoride were thereby identified and are described in detail, and the likely mechanisms of action were identified. The latter are based on the known aqueous chemistry of this substance, a topic that has been widely neglected in the dental literature.

## 2. Background

Since the beginning of the twentieth century, fluoride has been known to have anti-caries effects [1] and to reduce both the incidence and severity of dental caries [2]. In order to obtain these benefits, fluoride has been administered to patients in a variety of ways, including in drinking water, in toothpastes (also called dentifrices), in mouthrinses and as professionally applied drops [3,4,5]. In general, the main concern has been to provide an adequate supply of fluoride, and three compounds have been used widely as delivery substances, namely, sodium fluoride [6], sodium monofluorophosphate [7] and stannous fluoride [8]. The latter is the subject of this review, mainly its use as the source of fluoride in toothpastes.

According to one systematic review, concerns have been expressed in the literature that stannous fluoride in toothpaste might have negative health effects [8]. However, after analysing a range of published papers, and considering over 800 results from them, the authors concluded that these toothpastes present no important contraindications. Their general finding was that the use of stannous fluoride in toothpastes confers beneficial effects with no obvious drawbacks [8].

Fluoride in general and stannous fluoride in particular have various clinical effects. It arrests caries in the mineral phase of teeth [9], it reduces the viability of the oral biofilm known as dental plaque [10], it lessens dental hypersensitivity [11] and it improves gingival health [12]. Typically, stannous fluoride was found to be the most effective additive against all four of these conditions [13]. However, the mechanism of this effectiveness was rarely considered and, where it was, there were serious errors in the understanding of the solution chemistry of stannous fluoride [14,15,16]. In particular, several authors claimed incorrectly that stannous fluoride is a purely ionic compound and that it acts simply as a source of fluoride ions [14,15,16].

In fact, stannous fluoride is a largely covalent substance [17] and the clinical effects must be entirely due to the covalently bonded tin–fluorine species that have long been known to occur in aqueous solutions of SnF_2_ [18,19]. The current review aimed to relate the known clinical performance of stannous fluoride to its solution chemistry, and to draw out suggestions for its mechanism(s) of action that take account of the tin–fluorine species that are known to form in an aqueous solution. This review therefore includes some key studies that have been widely neglected in the dental literature.

## 3. Toothpaste

Toothpaste, also known as dentifrice, is an over-the-counter product designed to maintain the health and cleanliness of teeth. Used regularly and applied with the aid of a toothbrush, it is important in the promotion of oral health [8].

Toothpaste is a complex mixture of components, each with a specific role to play [20]. It is a high-viscosity paste, and the main components are water (20–40%) and an abrasive (50%), which may be a relatively simple substance, such as calcium carbonate or silica, or may be a bespoke synthetic mineral, depending on the brand. Other solid particulate components, such as calcium hydrogen phosphate or hydroxyapatite, may be included. Their function is to aid the remineralisation of the tooth mineral and to occlude the dentinal tubules to reduce or eliminate hypersensitivity.

Toothpastes also contain minor amounts of other components. These include detergent, typically sodium lauryl sulfate (0.5–2.0%), whose function is to promote the wetting of the tooth surface as an aid to cleaning [20]. Other components present in small amounts are humectants, which are hygroscopic substances that keep moisture in the toothpaste to prevent it from drying out. There are also usually traces of flavouring compounds, typically spearmint or peppermint. Depending on the formulation, there may also be substances added in small amounts to impart distinct clinical effects, such as against hypersensitivity (e.g., potassium nitrate) and/or to inhibit calculus formation at the tooth surface (e.g., sodium polyphosphate). Lastly, there may be a fluoride compound of some sort that was designed to reduce the incidence of caries.

The complexity of the formulation is necessary because toothpastes are required to perform a number of functions. As well as aiding in cleaning the tooth surface, they need to prevent caries, eliminate halitosis, inhibit gum disease, and remove traces of food and of dental plaque from the teeth [8]. Modern toothpastes not only perform these tasks to highly satisfactory extents, they perform the other tasks we note, such as protecting against caries and promoting remineralisation. They may also promote bleaching, at least to an extent [20].

Many brands of toothpaste provide a source of fluoride, a species that was shown in numerous studies to prevent dental caries [21,22]. Toothpaste is not the only possible source of fluoride for this purpose, but it is a highly effective one [21]. Compounds used are sodium fluoride [6], sodium monofluorophosphate [7] and stannous fluoride [8], and most studies concentrated on the fluoride component of these substances and ignored the effects of the counter-ions.

All three substances are known to be clinically effective at reducing the incidence of caries [8,20]. They are approved for use as additives in toothpaste by a variety of authorities, including the United States Food and Drugs Administration (FDA). Typical concentrations of fluoride in toothpastes are around 1000 ppm or a little above (up to 1500 ppm) [23], though much higher concentrations, i.e., 5000 ppm, are available by prescription for use by patients who are particularly susceptible to tooth decay [22].

## 4. Dental Caries and Fluoride

Dental caries, i.e., tooth decay, is considered to be the most common disease of humanity [8]. The condition is defined as “chronic, dietomechanical, site specific disease caused by a shift from protective factors favouring tooth remineralization to destructive factors leading to demineralization” [24]. The main factors that cause this shift are the presence of oral bacteria, principally *Streptococcus mutans*, as a component of the biofilm on the tooth surface, and fermentable carbohydrates in the diet of the patient [25]. This pairing leads to the production of organic acids adjacent to the tooth as a consequence of bacterial metabolism. The main acid is lactic acid, but other weak organic acids can also occur, including ethanoic (acetic) and propionic acids [26]. These compounds attack the mineral phase of the tooth, causing it to dissolve away and promote demineralisation [27,28]. Although these acids are weak in the sense defined by Bronsted–Lowry theory, they have a strong affinity for calcium ions, hence they readily attack the carbonate hydroxyapatite mineral phase of the tooth [27].

The part of the tooth affected by caries is the mineral phase. It consists of a naturally occurring version of the mineral hydroxyapatite with substitutions, mainly of carbonate [29,30]. This mineral is known as carbonate apatite.

The outer part of the tooth, the enamel, comprises some 97% of the carbonate apatite mineral [30]. This causes the enamel to be very hard. In fact, it is the hardest tissue in the body, and the hardness results not only from the chemical composition but also from the complex hierarchical arrangement of the needle-shaped crystals of carbonate apatite within the enamel [29,31,32]. The density of enamel lies between 2.6 and 2.8 g cm^−3^ [32], compared with 3.14 g cm^−3^ for pure crystalline hydroxyapatite [31]. Thus, enamel has a density that is about 80–85% that of the pure mineral, which indicates that the natural mineral has a degree of porosity [33].

The biological mineral differs in composition from pure hydroxyapatite. The main difference is that some phosphate groups are replaced by carbonate (CO_3_^2−^). In addition, a small proportion of phosphate groups are replaced by hydrogen phosphate (HPO_4_^2−^), and some of the calcium sites are vacant [34]. The hydroxide ion can also be replaced by fluoride, with consequences that are discussed shortly.

An acid attack of the mineral phase results in the development of a distinct cavity in the tooth. This cavity weakens the tooth mechanically and must be repaired in order to restore the tooth to a fully functioning state and to conserve it. The bacterial infection must also be removed to prevent further loss of the mineral phase of the tooth from the site of infection.

The loss of tooth structure in this way can be reversed in the process of remineralisation [34]. This can occur at the surface of the tooth by the diffusion of calcium and phosphate ions into the tooth mineral from the saliva. At the tooth surface, two things happen. The natural buffering effect of saliva shifts the pH away from the distinctly acidic pH, i.e., around 4.5 or lower, to a closer-to-neutral pH, i.e., 5.5 or higher [26]. In the latter condition, caries cease to be active and becomes arrested. As well as this, certain sites on the surfaces of the remaining tooth mineral act as nucleation sites and cause calcium and phosphate ions to precipitate as a fresh mineral phase [27]. The latter is driven by the fact that saliva is saturated with respect to both of these ions, which hence are present at high enough levels to remineralise the tooth surface [34].

Fluoride ions assist in combating the development of dental caries [2,35]. It appears to do so by three mechanisms, as follows:

(i) Formation of fluorapatite mineral [36,37,38,39,40], at least in thin layers [38], on the surface of the carbonate apatite phase. The fluorapatite mineral is less soluble than the carbonate apatite, and this reduces the rate of dissolution in the early stages of demineralisation [41].
(ii) Shifting the demineralisation–remineralisation steady state to favour the remineralisation processes [42,43,44].
(iii) Reducing the ability of saliva to solvate calcium and phosphate ions by forming strong hydrogen bonds with water molecules. This causes calcium and phosphate to be less soluble in the saliva, thereby promoting remineralisation [21,45].

## 5. Stannous Fluoride in Toothpaste

Studies on SnF_2_ as the fluoride component in toothpastes considered four aspects of behaviour. These were the effect on demineralisation, reducing hypersensitivity, the extent and viability of the plaque film, and the influence on gingival health. These are now considered separately. It should, however, be noted that most studies consider more than one of these aspects.

As well as including stannous fluoride in toothpastes, this substance has been added to mouthrinses, and several studies have been published on the effects of these fluoridated mouthrinses [46,47,48,49]. In the case of these mouthrinses, the main focus has been on the effect of the stannous fluoride on bacterial plaque.

Including stannous fluoride in mouthwashes is a less effective way of delivering it to the tooth surface. In toothpastes, the viscosity of the formulation is relatively high, and this helps keep the SnF_2_ close to the surface of the tooth. The interaction of the SnF_2_ at the surface is aided by the cleaning effect of the surfactant and the roughening effect of the abrasive particles. These cause the SnF_2_ to interact more efficiently with the mineral phase of the tooth than briefly covering the surface with mouthrinse containing SnF_2_.

## 6. Stannous Fluoride Toothpastes and Tooth Mineral

Stannous fluoride shifts the balance between demineralisation and remineralisation at the tooth surface. For example, in a study by Ganss et al. [50], it was shown that with a toothpaste that contained a combination of NaF, SnF_2_ and amine fluoride, demineralisation was reduced by 67%. By contrast, a control toothpaste that contained only NaF reduced it by only 19%. These results led to the suggestion that tin conferred additional protection by forming a layer of tin oxide/hydroxide that may be acid resistant [50].

Several other studies were carried out to determine the effect of stannous fluoride in toothpastes on the mineral phase of teeth. The toothpastes used were both commercial and experimental and, in addition to the individual studies, there was a systematic review published within recent years [8]. Typical findings of these studies are shown in Table 1.

The main conclusion from these investigations was that the presence of stannous fluoride in toothpastes reduces enamel loss due to caries. Moreover, where comparisons were made, stannous fluoride was generally shown to be more effective than either sodium fluoride or sodium monofluorophosphate.

Despite this, results typically attribute the effectiveness of stannous fluoride toothpastes to the effects of fluoride alone. Typically, no consideration has been given to the role of tin. In a typical investigation, carried out by West et al. [57], a stannous fluoride toothpaste was compared with one that contained sodium fluoride and triclosan, an antimicrobial compound. The results showed clearly that the stannous fluoride toothpaste gave greater protection against erosion to the enamel in the sample teeth. These observations were supported by those from other studies [7,51,53].

## 7. Stannous Fluoride Toothpastes and Dental Plaque

Several studies showed that stannous fluoride has antibacterial properties and can reduce the growth of plaque on the tooth surface. In this way, it can control the viability of the biofilm [8]. Findings from various recent studies are shown in Table 2.

The main conclusion from these and related studies [32,51,63,64] was that stannous fluoride in toothpastes reduces the viability of dental plaque to a statistically significant extent. As with the promotion of remineralisation, stannous fluoride is more effective than either sodium fluoride or sodium monofluorophosphate, suggesting that its activity is not simply due to the presence of fluoride. Some authors have attributed the antimicrobial effects to the presence of tin, specifically to Sn^2+^ ions [65,66], which, as we have seen, is based on a misunderstanding of the literature on the chemistry of tin in aqueous solutions. This is discussed in detail later.

In addition, the inclusion of stannous fluoride in mouthrinses was shown to reduce the viability of dental plaque [47]. No evidence was presented to show how this compared with other fluorides, such as NaF.

Another microbiological problem that occurs in patients is gingivitis, i.e., inflammation of the gums. Gingivitis is mainly caused by inflammation induced by bacteria from the dental plaque, so it is influenced by the health and extent of that plaque [67]. In a recent randomised clinical trial that involved twenty-four weeks’ use of an experimental toothpaste that contained 0.454% *w*/*w* stannous fluoride compared with a control toothpaste, there were statistically significant differences between the two toothpastes [68]. The use of the stannous fluoride led to the control of gingivitis and reductions in the levels of supra-gingival plaque. The study examined the level of bleeding sites as an indicator of clinical periodontal health, and the number of these sites was reduced substantially following 24 weeks of product use. This reduction correlated with less swelling of the gums and less plaque adjacent to the sites of swelling.

In a similar study carried out over a three-week period, when brushing twice a day with an experimental toothpaste that contained 0.454% *w*/*w* stannous fluoride compared with a control toothpaste, there were statistically significant differences between the two toothpastes [69]. The use of the stannous fluoride led to lower gingival bleeding, less gingival inflammation and reduced plaque levels in adults who had mild-to-moderate gingivitis. The results show that clinically beneficial reductions in plaque levels could be observed from two weeks of using stannous fluoride toothpaste, and that gingivitis declined as a result of these reductions.

In another randomised clinical trial, the extent of gingivitis was evaluated by determining bleeding when probed, the gingival index, and the plaque index after using either a stannous fluoride toothpaste stabilised with zinc phosphate or a sodium fluoride control toothpaste [70]. These groups were also compared for oral neutrophil counts, systemic priming of neutrophils, gingival crevicular fluid (GCF) expression of inflammatory markers and the oral microbiome. The results show that the stannous fluoride toothpaste led to a clinical reduction in gingival inflammation, a reduction in microbiome population and improvements in immune markers. These findings suggest that the use of stannous fluoride toothpaste has the potential to prevent plaque-induced gingivitis from progressing to full periodontitis.

The overall conclusion from these and other studies is that stannous fluoride in toothpaste has beneficial effects when treating gingivitis. Through its effects in reducing the viability of dental plaque, stannous fluoride removes, in part at least, the cause of gingival infection, thereby reducing localised swelling and bleeding of the gums.

## 8. Stannous Fluoride Toothpastes and Hypersensitivity

Over the years, there were a number of studies on the effect of stannous fluoride toothpaste on hypersensitivity. The results varied. Some studies concluded that the presence of SnF_2_ causes toothpastes to be effective at reducing hypersensitivity [58,71]. Others were unable to detect any differences between stannous fluoride toothpastes and additive-free controls [72,73,74].

An example of the former was the study by Hines et al. [71], in which a stannous fluoride toothpaste that contained 0.454% SnF_2_ was compared with a control toothpaste. The stannous fluoride toothpaste was found to lead to the tubules becoming occluded in in vitro studies and showed substantially less in vivo clinical sensitivity after 4- and 8-week treatments when tested with tactile and air-blast techniques [70].

However, these positive outcomes were not confirmed by other studies. For example, a randomised clinical study by Tao et al. [72] showed no differences in hypersensitivity with the use of a toothpaste that contained 0.454% SnF_2_ compared with toothpastes that contained either sodium monofluorophosphate or a blend of SnCl_2_ and NaF. All three toothpastes were found to reduce dentine hypersensitivity, and stannous fluoride conferred no clinical advantages. A similar study on a Chinese population confirmed this result [74]. The authors attributed their findings in part to the placebo effect, which they suggested rendered all three toothpastes equivalent in terms of their clinical effectiveness. Whatever the reason, these studies agreed in concluding that stannous fluoride was not effective against hypersensitivity.

These contradictory results make it difficult to decide whether stannous fluoride in toothpastes is beneficial against hypersensitivity. However, given that an important mechanism of reducing hypersensitivity is occluding the tubules, and that this process is assisted by remineralisation, stannous fluoride clearly has some benefit. Against this, several other additives are also beneficial and, depending on the details of the study, are equivalent in promoting occlusion of the tubules.

## 9. Aqueous Solutions of Stannous Fluoride

**Figure 1:**
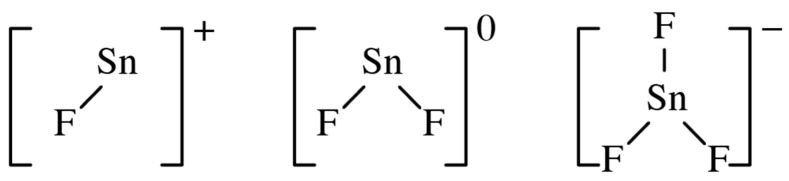
Tin(II) species found to occur in aqueous solution.

There were numerous studies of the chemical species that can form in aqueous solutions of SnF_2_. These mainly involved mixtures of tin(II) fluoride with other metal fluorides, such as ammonium, lithium or sodium fluoride. The structures of the tin–fluorine species found to occur in aqueous solution are shown in Figure 1, and the results of structural studies are shown in Table 3.

From the results in Table 3, it can be seen that the first such study was published in 1954 [75]. It used the largely obsolete technique of polarography and clearly identified the anion SnF_3_^−^. The authors also suggested that there was some evidence of the SnF_6_^2−^ ion, which they claimed was formed by the oxidation of Sn(II) to Sn(IV). However, there have been no other reports of this ion, so we have to conclude that it was either something else or arose from the use of perchlorate solution, which is oxidising.

The main species reported in all the other studies is SnF_3_^−^. This is formed by the reaction of excess fluoride ions from compounds such as NaF with undissociated SnF_2_. Depending on the ratio of compounds in the aqueous solutions studied, undissociated SnF_2_ may also be detected.

Other tin(II) fluorine species are known. For example, the compound Sn_4_OF_6_ was shown to be formed by the hydrolysis of SnF_2_ under appropriate conditions, and its structure was determined by X-ray diffraction [81]. Despite the fact that its formation clearly involves a reaction with oxygen, no oxidation of the tin occurred in its formation, and all four tin atoms remained in the +II oxidation state [80].

**Figure 2:**
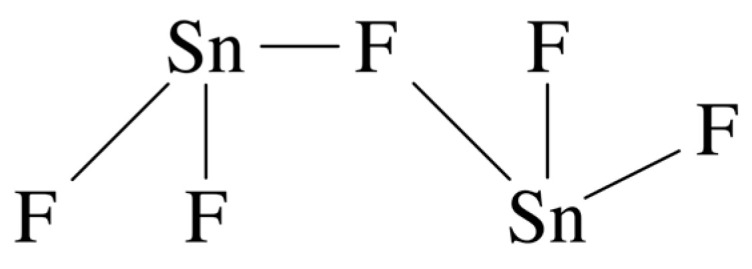
Bridging structure of the pentafluorostannate(II) ion [77].

Another known tin(II) ion is Sn_2_F_5_^−^, the pentafluorostannate(II) ion [83], which occurs in the solid state and has the structure illustrated in Figure 2. It contains a bridging fluorine atom with a F-Sn-F bond angle of 134.4°. Though well-characterised in the solid state, there is no evidence that it occurs in the liquid phase. It was not found in a study that involved an examination of appropriate molten tin(II) fluoride mixtures using infrared and ^119^Sn Mossbauer spectroscopy [84] and it has not been reported in aqueous solution either [77].

The main conclusion from the data in Table 3 is that SnF_2_ does not dissociate in aqueous solution in a simple way to give Sn^2+^ and F^−^ ions. Rather, over the short term at least, fluoride stays bound to tin, with SnF_3_^−^ being the principal fluorostannate(II) ion that forms in aqueous solution. This is a reflection of the high strength of the Sn(II)–fluorine bonds [80]. In the solid state, the SnF_3_^−^ ion is trigonal pyramidal, and the Sn-F bonds are short, i.e., 204 pm [85]. They were also shown to be covalent [86]. In fact, all of the common tin–fluorine species have short bonds, with linear decreases through SnF_2_ to SnF^+^, with the latter ion having the shortest Sn-F bonds, all being 198 pm long [85]. The extent to which these bond lengths change, if at all, in aqueous solution is not known; it seems unlikely that they change much.

In the case of the SnF_3_^−^ ion, Mossbauer parameters allowed for the nature of the electron pairs around the tin(II) ion to be determined. These parameters show that the fluorines bond to tin through three short bonds with a high s character at the tin. This s character was attributed to a combination of electrostatic and crystal field effects generated by the three fluorines [87]. The fourth electron pair is a non-bonding lone pair of high p character and with strongly directional properties [80].

These respective characteristics of the bonding and lone pairs arise because of the relative energies of the fluorine 2p and the tin 5s electrons. These relative energies mean that the tin–fluorine bonds must involve s orbitals on the tin rather than p orbitals. As a result, the non-bonding electrons on tin in the trifluorostannate(II) ion must have a high p character [80].

The high s character of the outermost tin electrons in SnF_3_^−^ make the resulting Sn-F bonds short and strong. This contrasts with the situation in the other possible trihalogenostannate (II) ions. In SnCl_3_^−^, for example, the binding energy of the chlorine p electrons lies between those of the tin 5s and 5p orbitals, with the result that tin(II)–chlorine bonds have a mixed s and p character. In turn, this makes them weaker than the equivalent fluorine bonds. Also, the lone pair occupies an orbital with a lower p character and reduced directionality compared with the one in the SnF_3_^−^ ion [88]. The net effect is that tin(II) fluoride and tin(II) chloride behave differently in aqueous solution.

Tin(II) chloride will dissolve in water, apparently without decomposing. In dilute solution, it is readily hydrolysed to an insoluble basic salt:

SnCl_2_(aq) + H_2_O(l) ⇌ Sn(OH)Cl(s) + HCl(aq)

To maintain SnCl_2_ in solution, hydrochloric acid must be added. Like SnF_2_, SnCl_2_ forms three distinct species in solution, namely, SnCl^+^, SnCl_2_ and SnCl_3_^−^ [89,90]. It also readily forms the hydroxide species Sn(OH)^+^, Sn(OH)_2_ and Sn(OH)_3_^−^, as well as mixed hydroxy-chloride species [32]_._ These hydroxide-for-chloride exchange products form more readily than the equivalent products of reaction with tin(II) fluoride. Both halides will undergo oxidation as a result of reaction with dissolved oxygen from the air, leading to the formation of Sn(IV) species [80,89], typically forming finely divided SnO_2_, which precipitates [90,91]. In order to prevent this, stannous fluoride toothpastes are generally stabilised with a substance such as zinc phosphate that will prevent the oxidation reaction from occurring.

## 10. Stabilisation and Speciation of Stannous Fluoride Toothpastes

When stannous fluoride is oxidised in aqueous systems, it forms SnO_2_, a substance that is abrasive when this occurs in a toothpaste such that the surface becomes readily roughened and stained [48]. This is undesirable, and consequently, such oxidation needs to be inhibited. This is achieved by careful formulation of the toothpaste, with one approach being to decrease the water content and another being to add a stabiliser, e.g., zinc phosphate [92,93].

The stability of SnF_2_ in toothpaste, and the species generated by oxidation, were studied using X-ray Absorption Spectroscopy (XAS) [91]. This is an element-selective technique that needs only minimal sample preparation. It allows for the speciation of metals to be determined in a variety of matrices, including viscous media, such as toothpastes. The results for the study of SnF_2_ in toothpaste are summarised in Table 4.

These results show that stannous fluoride in all four toothpastes underwent a degree of oxidation during packing and storage, with toothpaste 4 being particularly affected in the early stages. In the case of toothpastes 1 to 3, there was further oxidation after the tubes had been opened a number of times due to the uptake of fresh oxygen from the air and further reaction to form Sn(IV) species [91].

Further analysis of the XAS spectra of the toothpastes gave additional information about the tin species present. The unopened tubes were found to contain some sort of Sn(II)-O species with a modified tetrahedral structure. The remaining tin was present as an Sn(IV)-O species in an octahedral co-ordination. According to the authors, the latter seemed most likely to be some sort of stannic hydroxide species, such as Sn(OH)_4_ [32].

Surprisingly, there was no spectroscopic evidence for the occurrence of SnF_2_ [91]. This suggests that although much of the tin remained in the +II oxidation state, it had undergone ligand exchange processes and converted to a predominantly Sn(II)-O species. Some of the tin was clearly detected as a Sn(II)-O-P species, which may be why it does not oxidise further to Sn(IV). The formation of such species appears to be the mechanism of stabilisation of the Sn(II) oxidation state by the addition of phosphate, e.g., of zinc, to the toothpaste formulation [92].

These results indicate how complex the chemistry of stannous fluoride is in toothpastes. They also indicate the type of species that need to be considered when determining how stannous fluoride acts against dental caries.

## 11. Mechanism of Action of Stannous Fluoride

In aqueous solution, SnF_2_ exists predominantly in an undissociated form [82]. The equilibrium constant for the process

SnF_2_ ⇌ SnF^+^ + F^−^

was measured and found to be 8.8 × 10^−5^ mol dm^−3^. In other words, the predominate species that occurs when stannous fluoride dissolves in water is undissociated SnF_2_. In the hydrated state, there is evidence that a water molecule becomes co-ordinated to the SnF_2_ molecule via a tin–oxygen bond [85].

The results reported in the previous section show that SnF_2_ reacts within a toothpaste to form oxygenated Sn(II) species. In doing so, it almost certainly generates free fluoride ions. However, the incorporation of SnF_2_ produces a toothpaste that is more effective against dental caries than other fluoride compounds [50,51,52,53,54,55,56], and this shows that, in the words of Faller and Noble, all fluorides are not equal [93]. Tin in the +II oxidation state also plays a part in the effects of employing SnF_2_. Consequently, when considering the mechanism of action, we must consider the effects of both the fluoride ions and the tin(II) species.

An important feature of Sn(II) is that it is “hard” in Pearson’s Hard and Soft Acids and Bases scheme [94]. This means that it forms its strongest bonds with, and has the highest affinity for, elements such as oxygen and fluorine. Tin(II)–fluorine bonds have a mean bond enthalpy of 467 kJ mol^−1^ [95], whereas tin(II)–oxygen bonds are even stronger, with a mean bond enthalpy of 548 kJ mol^−1^ [95]. These values explain the high stability of the Sn(II)-O species in aqueous solution.

The fluoride ion is small, which is indicated by its ionic radius of 133 pm [96]. The hydroxide ion is even smaller, with an ionic radius of 110 pm [97]. This feature, together with the higher bond strength of the Sn(II)-O bond compared with the Sn(II)-F bond, implies that the Sn(II)-OH species will form preferentially in aqueous solutions of SnF_2_, and it will be stable when it has formed. This, in turn, suggests that the eventual species that occur in aqueous solution are Sn(OH)_2_ and Sn(OH)^−^. These are the expected products of the reactions of SnF_2_ and SnF^−^, respectively. When considering the mechanism of action of toothpastes initially formulated with SnF_2_, it is these species that we must consider.

As shown earlier in this article, one of the means of protecting the teeth from caries is to reduce the population of micro-organisms, notably *Streptococcus mutans*, in the biofilm. The use of stannous fluoride has been known for over fifty years to provide clinical antimicrobial effects [98]. In addition, including stannous fluoride in toothpaste is known to supply chemical species that damage *S. mutans*, and thereby reduce the caries activity of the biofilm. The hydroxide-for-fluoride exchange, which was shown to occur in the toothpaste in storage [91], must liberate fluoride ions, and these are known to be toxic towards micro-organisms [99]. Fluoride toxicity appears to involve four distinct processes, namely, the inhibition of proteins, the disruption of organelles, the alteration of pH and unbalancing the electrolyte concentrations [99]. In creating these effects, stannous fluoride acts simply as any other of the fluorides used in toothpastes as an anti-caries agent.

However, there is an additional effect from the Sn(II) component. As was shown, this is not due to it being present as simple Sn^2+^ ions, but rather because it forms some sort of strongly bonded hydroxide species, either Sn(OH)^+^ or Sn(OH)_2_. If sodium fluoride is also included in the formulation, SnF_3_^−^ ions form initially in the toothpaste mixture, and these can undergo conversion to Sn(OH)_3_^−^. All three of these hydroxides, by analogy with the possible fluorides, are capable of forming a strong co-ordinate bond to oxygen atoms in complexes.

One important target compound is glycosyl transferase, an enzyme present in *S. mutans* [100]. It is a useful target for reducing the activity of *S. mutans* in the biofilm and thereby inhibiting the development of dental caries. It also contains numerous oxygen atoms, which can act as targets for bonding to Sn(II) species, and in this way, its action can be inhibited. *S. mutans* uses this enzyme to convert sucrose to an extracellular glucan polymer that causes the biofilm to adhere to the tooth surface. This promotes the build-up of the biofilm [101]. Targeting this enzyme reduces the biofilm build-up, which, in turn, reduces the extent of dental caries [100].

As well as having a toxic effect on *S. mutans*, stannous fluoride was shown to interact with hydroxyapatite or natural tooth mineral and alter its susceptibility to dissolution or erosion [102,103]. Two papers have reported that treating teeth with solutions containing Sn(II) species and fluoride ions reduced erosion in vitro [102,103]. Both show that tin was taken up by the mineral phase. Unfortunately, they made the mistake of describing the tin species as Sn^2+^ ions, which, as has been demonstrated, is incorrect.

There were studies of tin uptake by synthetic hydroxyapatite when this mineral is exposed to aqueous SnF_2_ solution. Many years ago, the product of reaction was found to be the crystalline compound Sn_3_PO_4_F_3_ [104]. A few years later, another study found that the compounds Sn_2_(OH)PO_4_ and Ca(SnF_3_)_2_ were also formed [105].

Synthetic hydroxyapatite does not completely match either the composition or the structure of natural hydroxyapatite as found in teeth [106,107,108]. As we have seen, natural hydroxyapatite is non-stoichiometric and has up to 8% carbonate substitutions [108,109] compared with the synthetic mineral. These substitutions make the natural mineral less crystalline and more soluble than the synthetic version [106,107,108,109,110]. However, the two minerals resemble each other sufficiently closely for synthetic hydroxyapatite to be used to model the natural mineral in in vitro experiments.

Studies confirmed that synthetic hydroxyapatite takes up tin from aqueous SnF_2_ solutions [83,84]. One of these studies used ^119^Sn Mossbauer spectroscopy to examine the nature of the tin taken up [80]. It showed that the predominant species present on the hydroxyapatite was Sn(II), though some Sn(IV) was also detected [81]. Peak splitting in the Mossbauer spectrum showed that the Sn(II) was covalently bound to fluorine, suggesting that either SnF_2_ or SnF^+^ was taken up. Tin uptake was confirmed by EDAX in scanning electron microscopy [82], and this latter study used results from fluoride determination with an ion-selective electrode to infer that the species involved was SnF^+^. These uptake studies thus gave comparable findings.

The latter paper reported uptake from solutions at two different concentrations, namely, 500 and 1000 ppm in fluoride [82]. The results show that the hydroxyapatite powder adsorbed the same amount of fluoride to within experimental error, i.e., 11.3 mg/g and 11.5 mg/g for the 500 and 1000 ppm solutions respectively. This led to the suggestion that the hydroxyapatite powder had a clearly defined number of sites in the surface where the SnF^+^ could be taken up, and that both solutions provided more than enough SnF^+^ to occupy these sites [82].

Studies of toothpastes showed that SnF_2_ is converted into Sn(OH)_2_ over the time that the toothpaste is in the tube. This shows that the species involved when such toothpastes are used are hydroxides rather than fluorides. However, this is likely to make little difference to the adsorption process. The result is still likely to be a bonded Sn(II) species, either Sn(OH)_2_ or Sn(OH)^+^, with the latter being more likely. Whether such a species goes on to form a well-defined compound, such as Sn_3_PO_4_(OH)_3_, analogous to Sn_3_PO_4_F_3_, is not clear. However, what is likely is that, even after ageing, the use of such toothpastes probably results in the uptake of a tin(II) species and this does not consist of Sn^2+^ ions. The uptake of this Sn(II) species probably produces a modified surface layer that is more resistant to dissolution than the native mineral phase, and thereby enhances remineralisation. In this way, the Sn(II) species modifies the hydroxyapatite such that it resists dental caries.

## 12. Conclusions

This literature survey showed that including stannous fluoride in toothpaste imparts clinically beneficial properties. In particular, stannous fluoride promotes antimicrobial and anti-caries behaviour. This behaviour is superior to that provided by other fluorides, such sodium fluoride and sodium metafluorophosphate, and shows that the tin(II) species leads to enhanced properties.

The speciation of the tin(II) component has been discussed in detail. Several clinical papers have attributed the effects to the occurrence of Sn^2+^ ions but, as numerous studies demonstrated, free Sn^2+^ ions do not form in aqueous systems. Rather, over shorter timescales, tin(II)–fluorine species occur in which fluorine is bonded to Sn(II) with strong and reasonably stable Sn-F bonds. Over longer time periods, however, in toothpastes, SnF_2_ and SnF^+^ become converted into Sn(OH)_2_ and Sn(OH)^+^, and these species interact with oral bacteria, mainly *S. mutans*, to reduce their viability in the oral biofilm. They also interact with the natural hydroxyapatite of the tooth mineral to form thin films of tin-doped hydroxyapatite. This resists dissolution and enhances remineralisation. The formation of tin–hydroxide species liberates free fluoride ions into the aqueous phase, and these are toxic to oral bacteria and able to interact with hydroxyapatite to protect tooth mineral from caries. Hence, they also contribute to the anti-caries effects of stannous fluoride.

## Figures and Tables

**Table 1:** Typical findings of the effect of SnF_2_ toothpastes on tooth mineral.

Toothpaste Groups	Sample	Time/Days	Results	Reference
Four including SnF_2_	168 enamel surfaces	14	SnF_2_ gave greater remineralisation	[51]
Two, one SnF_2_ + NaF	33 enamel surfaces	15	SnF_2_ reduced enamel loss	[52]
Two SnF_2_ (0.4 and 0.454%)	64 human teeth	9	Both reduced enamel wear	[53]
One SnF_2_	27 enamel surfaces	17	SnF_2_ reduced plaque	[54]
Four including SnF_2_	16 human molars	9	SnF_2_ no different	[55]
Three, two with SnF_2_	20 enamel surfaces	5	SnF_2_ better than NaF	[56]

**Table 2:** The effects of SnF_2_ toothpastes on dental plaque/oral biofilms.

Toothpaste Groups	Time	Results	Reference
SnF_2_, SnF_2_ + zinc lactate, F	6 months	SnF_2_ reduced plaque	[58]
SnF_2_ and NaF	6 months	SnF_2_ reduced plaque	[59]
SnF_2_ and NaMFP	8 weeks	SnF_2_ reduced plaque	[60]
Zn-HAP and amine fluoride + SnF_2_	12 weeks	SnF_2_ reduced plaque	[61]
SnF_2_ and NaF	17 days	SnF_2_ same as NaF	[62]

**Table 3:** Tin (II)–fluoride species identified in aqueous solutions.

Species Identified	Additional Species Proposed	Technique	Reference	Year Published
SnF_3_^−^	SnF_6_^2−^	Polarography	[75]	1954
SnF_3_^−^	-	IR and Raman	[76]	1962
SnF_3_^−^	-	IR and ^119^Sn nmr	[77]	1965
SnF^+^, SnF_2_, SnF_3_^−^	F^−^	Fluoride electrode	[78]	1968
SnF^+^, SnF_2_, SnF_3_^−^	-	Polarography	[79]	1970
SnF_2_, SnF_3_^−^	-	^119^Sn nmr, ^19^F nmr and ^119^Sn Mossbauer	[19]	1984
Sn_4_OF_6_	-	Hydrolysis of SnF_2_	[80]	1994
SnF_2_, SnF_3_^−^	-	^119^Sn Mossbauer	[81]	1994
F^−^, SnF_2_	SnF^+^	Fluoride electrode	[82]	2013

**Table 4:** Sn(II) species (%) in four toothpaste samples [91].

Toothpaste Number	Unopened	Opened
1	85 ± 1	62 ± 1
2	78 ± 1	60 ± 2
3	87 ± 1	61 ± 1
4	67 ± 1	67 ± 1

## Data Availability

No new data were created for this article and full references are provided for all the previously published data described and discussed in this article.
